# Benign Early Repolarization Phenomenon in Young Adults and Athletes: A Literature Review of Clinical Insights and Diagnostic Considerations

**DOI:** 10.7759/cureus.90581

**Published:** 2025-08-20

**Authors:** Alberto Rojas Peláez, Enmanuel Sevilla Torres, Daniela Fernandez Vinocour, Freddy Lizano Guevara, David Sáenz Araya

**Affiliations:** 1 General Medicine, Universidad de Ciencias Médicas (UCIMED), San José, CRI

**Keywords:** holter monitoring, j-point elevation, risk stratification, st-segment morphology, sudden cardiac death, ventricular arrhythmias

## Abstract

Early repolarization (ER) is an electrocardiographic pattern characterized by J-point elevation and ST-segment changes, historically considered a benign finding, especially in young adults and athletes. This review explores the clinical relevance of ER, emphasizing its electrophysiological basis, demographic patterns, and implications for risk stratification. While ER frequently appears in healthy individuals, particularly those engaged in high-intensity training, certain morphological variants and dynamic patterns have been associated with an increased risk of life-threatening arrhythmias and sudden cardiac death (SCD).

The prevalence of ER is notably higher in athletes, reflecting physiological adaptations to sustained physical activity rather than underlying pathology. However, in middle-aged individuals or those with a personal or family history of arrhythmias, time-varying ER may suggest a more arrhythmogenic substrate requiring closer surveillance.

Electrocardiographic features such as horizontal ST segments, fragmented QRS complexes, or widespread J-point elevation may indicate a higher risk. Comprehensive diagnostic evaluation includes history-taking, physical examination, Holter monitoring, exercise testing, and, when indicated, advanced imaging or electrophysiological studies. Management strategies emphasize contextual interpretation, shared decision-making, and personalized follow-up, particularly in athletes.

## Introduction and background

Early repolarization (ER) refers to distinct electrocardiographic findings and is characterized by the presence of a J-wave or J-point elevation, which is identified in the ST segment of the electrocardiogram (ECG) trace. ER can occur in a range of morphologies, but when seen, it is commonly seen in the inferior and lateral leads on a standard ECG (DII, DIII, aVf, V4-6) [1]. ER will typically include an ST segment that rises rapidly and a notching morphology on a lead that dips back below baseline at the end of the QRS complex [2].

ER has traditionally been treated as a benign ECG variant that is commonly seen, particularly in young active individuals [3]. This is due to ER having been found to occur in a limited compartment of asymptomatic individuals without evidence of structural heart disease. However, other types of ER have emerged that suggest acute repolarization may pose a greater risk for malignant ventricular arrhythmias and sudden cardiac death (SCD). These observations have led to a renewed scrutiny of its clinical relevance, especially in those with a personal or familial history of cardiac events [4]. Despite these evolving concerns, several longitudinal studies have not found a consistent association between ER and increased mortality in otherwise healthy individuals, suggesting that the risk may be limited to specific subtypes or clinical contexts [5].

The significance of ER is particularly noteworthy in athletes and young individuals, where it appears more frequently compared to their sedentary counterparts. Evidence indicates that the prevalence of ER is approximately 50% higher in athletes, reflecting potential physiological adaptations to intensive training rather than pathological alterations [3]. In pediatric athletes, ER has been documented as a common and generally benign phenomenon related to cardiovascular remodeling induced by sustained exercise. Longitudinal follow-up over four years has revealed no incidence of SCD in this group, further supporting its benign nature in this demographic [2]. Moreover, in adolescent athletes, ER prevalence demonstrates gender-related differences and correlates with distinct electrocardiographic and echocardiographic profiles, underscoring the importance of contextualizing ER within broader physiological parameters [6].

ER is a common incidental finding during routine cardiovascular testing and is commonly identified among younger adult individuals. While it is frequently an innocent finding very commonly observed, it does have an uncertain future as to represents an early marker for emerging cardiomyopathies or the emergence of substrates embodied by early arrhythmogenic pathways. At present, evidence is not sufficient to clearly identify the predictive value; however, the topic continues to be evaluated for potential outcomes in future prospective studies [7].

Given the fact that limited data exists around this topic, many clinicians will present a lack of knowledge when presented with ECG representations of benign ER in young, active, and healthy patients. This lack of awareness may result in augmented testing on otherwise healthy individuals, resulting in elevated unnecessary time and financial costs both for the patient and the healthcare system [2]. This review aims to raise awareness and provide up-to-date information on benign ER phenomena, with a particular focus on their prevalence, electrocardiographic features, and clinical relevance in young adults and athletes. 

## Review

Methods

The narrative review was constructed with a focused but flexible search strategy intended to provide a more comprehensive synthesis of the current understanding of benign ER phenomena, and specifically, that observed in young adults and athletes. For both breadth and depth of literature searched, the review utilized three major biomedical databases: PubMed, Scopus, and ScienceDirect. These databases provide substantial global coverage of research in published peer-reviewed medical and scientific research.

The search strategy limited publications to the previous five years (2020-2025) to allow for the research to be as up to date as possible in terms of clinically relevant data. The language of study was limited to English and Spanish publications to allow for reading comprehension and language accuracy. A combination of Medical Subject Headings (MeSH) and free-text descriptors was used, including the following keywords: J-point elevation, ST-segment morphology, ventricular arrhythmias, risk stratification, Holter monitoring, and sudden cardiac death. Boolean operators were applied to refine the search strategy to retrieve study results with clinical, pathophysiological, and electrophysiological viewpoints of ER.

Since rigid systematic criteria may inadvertently exclude valuable, recent, contemporary, and clinically appropriate literature, a flexible approach for the selection process was adopted. Studies were chosen based on relevance, quality, and applicability for clinical practice rather than strictly based on design. The literature selected will just include sources that are original research articles, systematic reviews, meta-analyses, and expert consensus statements published by reputable scientific societies and journals. Studies that were duplicates, not applicable clinically, or did not calculate outcomes for the study's population of interest were filtered out.

Studies that, despite their scientific validity, did not provide information directly relevant or transferable to clinical practice were excluded. This included research limited to laboratory or animal models without a clear link to patient care, studies conducted in populations or settings that differ substantially from the focus of the review, being the mean general population, or findings that could not be meaningfully incorporated into clinical decision-making. Only evidence offering practical and applicable insights for everyday medical practice was retained. 

**Figure 1:**
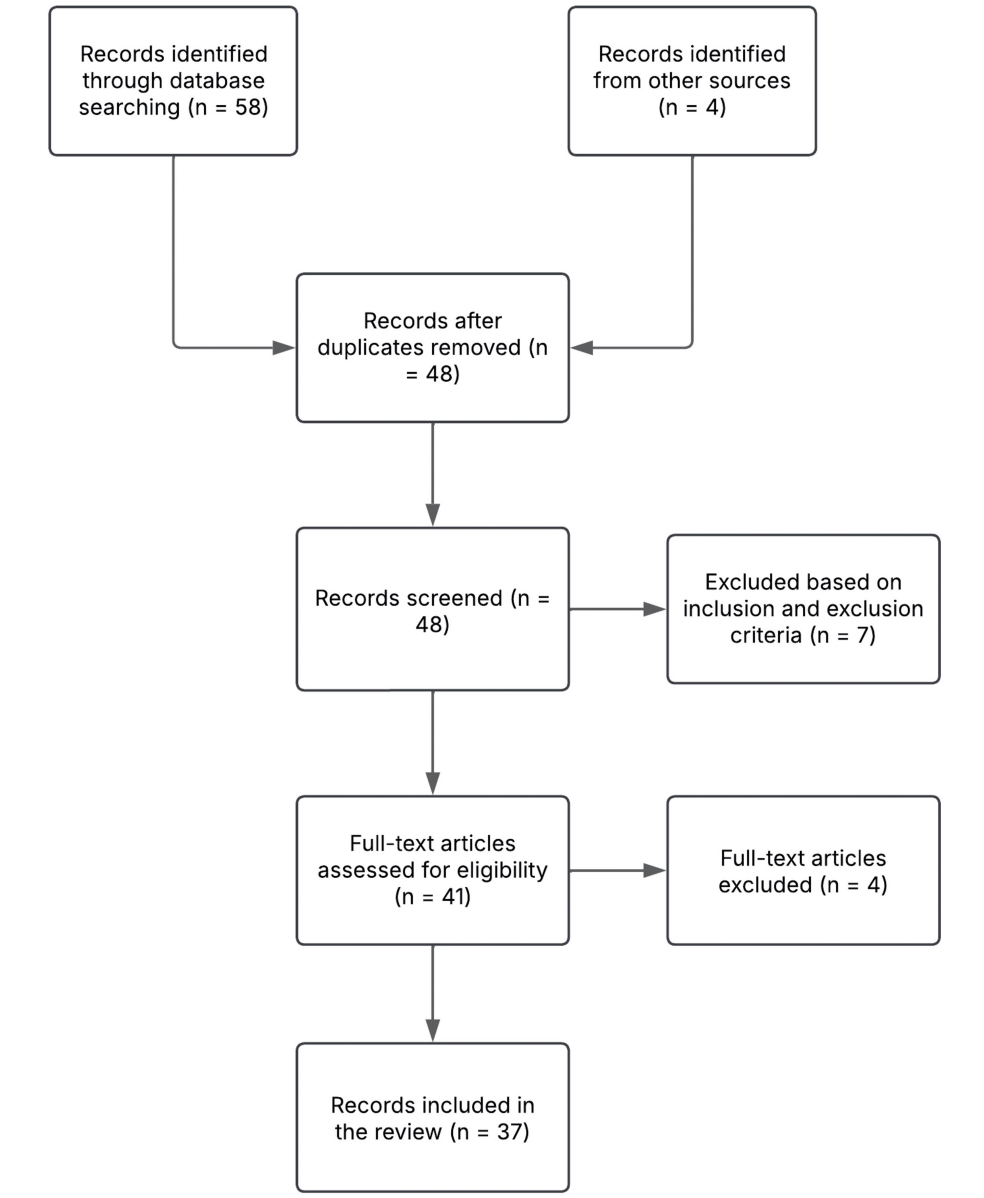
Flow diagram of the study selection process for the literature review on the benign early repolarization phenomenon in young adults and athletes

The compositions of the literature drew upon several observational studies, narrative reviews, ECG studies, experimental models, and position papers, which allowed the authors to consider a broader and more critical perspective on benign ER in the context of cardiac adaptation to exercise, risk stratification, and differentiation from malignant variants. Figure 1 summarizes the study selection process.

Finally, the authors used OpenAI's (OpenAI, Inc., San Francisco, California, USA) artificial intelligence (AI) tool to assist in the structural organization and linguistic refinement of the manuscript. While AI-assisted editing contributed to clarity and cohesion, all interpretative decisions, content validation, and scientific judgment were performed exclusively by the authors to preserve the academic and clinical rigor of the work.

Electrophysiological basis of early repolarization

The cardiac action potential is an essential electrophysiological activity that allows the heart to contract rhythmically, classically divided into five consecutive phases, phases 0 to 4. Phase 3, or repolarization, of the cardiac action potential is predominantly driven by the efflux of potassium ions, which return the membrane potential to a resting state following depolarization. The differences in action potential duration (APD), or the length of time that the action potential is present, in different areas of the myocardial wall create a natural heterogeneity in repolarization. These differences in repolarization seem to affect the effective refractory period (ERP), and the differences in the ERP play an important role in the electrical stability of the heart. These differences likely play an important role in the susceptibility of the human heart to arrhythmias [8].

One of the electrocardiographic phenomena associated with electrophysiological variability is J-point elevation, which is mostly seen in ER patterns. The J-point is an electrocardiographic mark indicating the transition from the QRS complex to the ST-segment, and the J-point can be described as elevated because the repolarization process produces transmural repolarization, or heterogeneity of the electrical potential, which originates from across the myocardium. Essentially, J-point elevation can be accentuated by a premature atrial contraction, resulting in the electrical potential being redistributed, which creates the appearance of a J-wave, the most dystonic aspect of an ER pattern. Another example can lead to pathological conditions related to repolarization, such as long QT syndrome, which can further exaggerate transmural dispersion of repolarization and subsequently lead to serious and potentially fatal polymorphic ventricular arrhythmias, such as Torsade de Pointes [9].

The principle of regional heterogeneity in repolarization goes beyond these available mechanisms and recognizes that APD can also vary not only through the endocardial and epicardial layers but also with respect to the apex-base axis of the ventricles [8]. This physiological heterogeneity becomes clinically relevant when it allows for reentrant arrhythmias to develop, especially in hearts with structural abnormalities or scars due to fibrosis. Recent advances in electrophysiological imaging techniques, particularly high-density repolarization mapping, have already proven valuable in identifying and localizing areas of electrical heterogeneity. Moreover, these techniques are increasingly used in the contexts of directing targeted therapeutic measures, such as ablation, in patients with complex arrhythmogenic profiles [10].

Epidemiology and demographics

ER is a common electrocardiographic pattern, frequently observed in both general and specific populations. In a large cohort study, ER was associated with an increased risk of SCD and cardiovascular mortality, highlighting its potential prognostic value even in asymptomatic individuals [11].

In pediatric athletes, Geza et al. found ER has been reported in 13.2% of cases (p = 0.072), suggesting that it is also a frequent finding in younger individuals. Notably, follow-up data in this group showed a benign clinical course, reinforcing the notion that the significance of ER may vary with age. Younger individuals, especially athletes, tend to exhibit ER more commonly, and its interpretation must consider the physiological adaptations linked to physical training [2].

Sex-related differences further influence the clinical understanding of ER. Males have been shown to have a higher incidence of SCD and cardiac arrest compared to females, which may affect how ER patterns are assessed in clinical practice [11].

Athletes, in general, demonstrate a higher prevalence of ER compared to non-athletes. Claessen et al. found that 24% of athletes had ER (OR 1.5 (SE 0.34), adjusted 95% CI 1.0 to 2.4), a rate significantly greater than that seen in the general population. Interestingly, this higher prevalence did not correlate with the type of sport or training duration, suggesting a broader physiological basis [3]. In adolescent athletes, ER presented with gender-specific ECG features, although overall prevalence did not differ significantly between sexes [6].

Ethnicity and genetic predisposition are also important contributors. Black athletes have shown a higher prevalence of ER and other ECG variants compared to Caucasian athletes, raising the need for ethnicity-informed interpretation standards [12,13]. Furthermore, genetic studies have identified mutations in calcium channel genes associated with ER syndrome, which may increase susceptibility to arrhythmias and SCD in certain individuals [14].

Electrocardiographic features

The electrocardiographic characteristics of ER show certain features that, while usually harmless, might be linked to arrhythmias in some clinical situations. One of the main signs of ER is J-point elevation, which refers to a small upward deflection at the point where the QRS complex ends and the ST segment begins. This is often seen in healthy individuals, especially young adults and athletes, but in some cases or populations, it has been connected to a higher risk of arrhythmias [1].

Another common feature is the appearance of slurred or notched terminal QRS complexes. These typically show up along with J-point elevation, although they were already being described before the concept of J-point elevation was clearly established. They’ve generally been viewed as harmless, but more recently, there's been interest in whether they could be linked to arrhythmogenic risk, especially if they’re seen along with other abnormal ECG findings [15].

**Figure 2:**
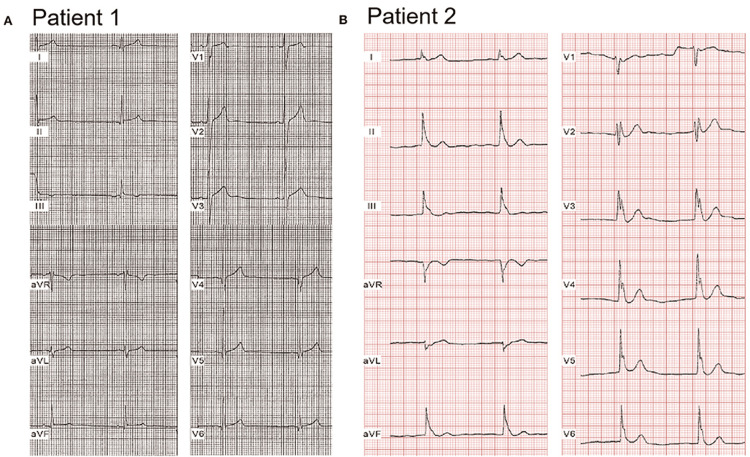
Representative electrocardiograms of two young patients with genetically confirmed early repolarization syndrome (ERS) A: Patient 1 was a 20-year-old male patient, identified with a mutation. He suffered syncopal episodes twice while exercising. ECGs showed bradycardia (HR = 46 bpm) and an ERP with notching in the inferior leads, leading to a diagnosis of ERS. B: Patient 2, a 17-year-old young male patient, presented with spontaneous VF on the background of syncope. ECGs exhibited sinus bradycardia (HR = 50 bpm) and an ERP with notching/slurring in a global pattern, thus diagnosed with ERS. Figure reproduced from Chen et al. licensed under CC-BY 4.0 [14]. ERP: effective refractory period; ERS: early repolarization syndrome; VF: ventricular fibrillation

Figure 2 illustrates two representative ECGs from young patients with genetically confirmed early repolarization syndrome (ERS). Both demonstrate sinus bradycardia and distinct J-point notching or slurring patterns. Patient 1, with ERS type 2, exhibited inferior lead notching, while Patient 2, with ERS type 3, presented a global ERP distribution. These cases emphasize the diagnostic importance of recognizing morphological variations in ERP and their potential link to genetic predisposition and arrhythmogenic risk [14].

Also worth mentioning is ST-segment elevation, particularly in the inferior and lateral leads. In many cases, this is just a normal variant seen in ER, but it’s also been described in conditions like Takotsubo syndrome or as a secondary repolarization change in situations such as right bundle branch block (RBBB). Because of that, its significance depends a lot on the clinical context [16,17].

**Figure 3:**
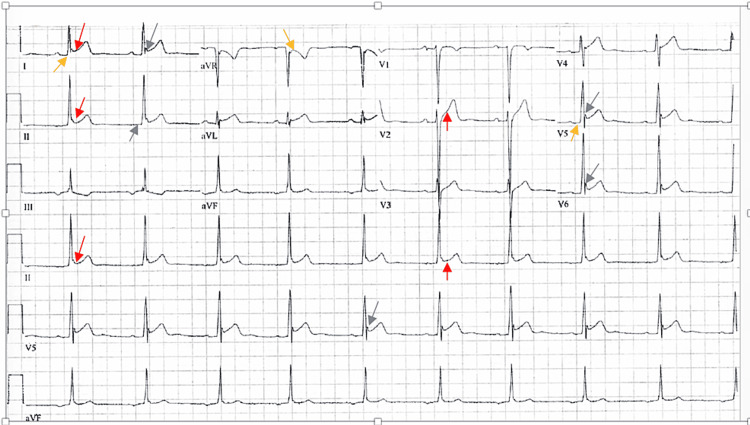
ECG from a patient with acute pericarditis Diffuse ST-segment elevations (red arrows), PR-segment depressions (orange arrows), “fish-hook” morphology (gray arrows), and T-wave inversion in lead III are observed. Figure reproduced from Amoateng et al., licensed under CC-BY 4.0 [18].

It is also essential to distinguish ER from other causes of ST-segment elevation, such as acute pericarditis (Figure 3), which can present with diffuse ST-segment elevations, PR-segment depressions, and characteristic “fish-hook” patterns. These features are often accompanied by T-wave inversions and help guide differential diagnosis based on ECG morphology and clinical presentation [18].

There are some proposed ways to tell the difference between a benign and a more malignant form of ER. The benign kind is usually seen in healthy young patients with no structural heart disease. It tends to be stable over time and not related to bad outcomes [5]. On the other hand, malignant ER has been linked to higher chances of ventricular arrhythmias and SCD. A key clue in these cases is the presence of fragmented QRS complexes (fQRS), which are considered strong predictors of major adverse cardiac events (MACE) [15].

**Figure 4:**
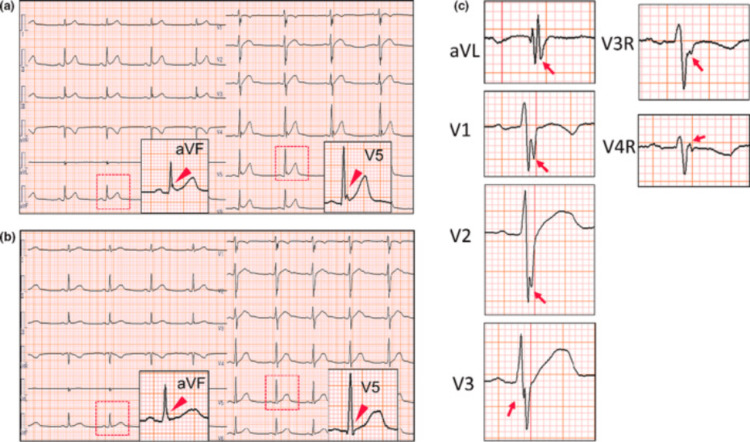
J-wave dynamicity and fragmented QRS on 12-lead ECG a: ECG showing accentuation of early repolarization in inferior and lateral leads (red arrowheads); b: ECG without ER pattern (red arrowheads); c: fragmented QRS (fQRS) seen in anterior (V1–3), lateral (aVL), and right precordial (V3–4R) leads (red arrows). Figure reproduced from Yonezu et al., licensed under CC BY-NC-ND 4.0 [20]. ER: early repolarization

This concept is illustrated in Figure 4, where Figure 4a shows clear augmentation of J-waves in inferior and lateral leads (consistent with a dynamic ER pattern), and Figure 4b demonstrates resolution of ER features. Additionally, Figure 4c highlights fragmented QRS complexes (fQRS) identified in anterior, lateral, and right precordial leads. The combined presence of dynamic J-wave behavior and fQRS may indicate higher arrhythmic potential [19].

That said, it’s important to keep in mind that other heart conditions can look similar on the ECG. For example, Takotsubo syndrome is a temporary heart condition triggered by stress that can cause ST-segment elevations, sometimes looking like an ST-elevation myocardial infarction (STEMI), but it usually resolves on its own without lasting damage [16]. And in patients who already have RBBB, ST-elevation in the same leads is often considered a normal finding, due to the way the electrical signal moves. But in cases where those expected changes are missing, it might actually point to something more serious [17].

Another condition to consider is arrhythmogenic left ventricular cardiomyopathy (ALVC). It can resemble ER on the ECG, but it usually shows T-wave inversion and low-voltage QRS complexes, and unlike typical ER, it reflects real structural problems in the heart and carries a higher risk of arrhythmias [20].

Pathophysiological considerations

ER tends to show up with certain ECG features, mainly J-point elevation and ST-segment elevation, and these are influenced by the function of different ion channels in the heart. Some of the most important ones are the L-type calcium channels and the HCN4 channels, both of which are involved in repolarization. Interestingly, in athletes, researchers have observed a reduction in these channels' expression, which could help explain why ER patterns are more commonly seen in this group [21].

One of the underlying mechanisms that could lead to arrhythmias in people with ER is the spatial dispersion of repolarization. This means that different areas of the heart repolarize at different times. That unevenness can create conditions where reentrant arrhythmias become more likely, especially if there are also changes in heart structure or in the way the nervous system regulates the heart [22].

Athletes also tend to have a different autonomic profile, with more vagal tone than average. That stronger vagal influence can affect how the heart conducts electricity; it often leads to longer PR intervals or even atrioventricular (AV) blocks, which are usually harmless in well-trained individuals [20]. But the autonomic nervous system (ANS) doesn’t just affect conduction; it also interacts directly with ion channels, and in some patients with ERS, sudden increases in vagal activity have actually been linked to arrhythmias [10].

Although ER is usually considered a benign pattern, especially in athletes, not every ER case can be classified as such. Certain types, like new-onset ER or ER that changes over time, have been linked to higher chances of SCD or cardiovascular mortality, especially in older untrained individuals [11].

In comparison, studies on pediatric athletes show a much safer picture. ER was common in those kids, but no major heart events were recorded in the follow-up periods. This suggests that age, and probably the heart’s adaptations to exercise, change the meaning of ER and make it less concerning in that population [2].

All of this underlines why it's so important to figure out whether an ER finding is benign or not, especially in athletes. Misinterpreting the pattern can lead to overreactions such as unnecessary tests and procedures, and ultimately keep a healthy athlete from competing. On the other hand, a benign ER diagnosis has to be thorough enough not to let a more serious condition go by undiagnosed. Some ECG details, like how big the J-wave is or where it shows up, have been suggested as helpful for figuring out which cases need closer follow-up [3,23].

Clinical implications and risk stratification

Trying to tell the difference between benign and malignant forms of ER is still a real issue in clinical practice, especially in young adults and athletes, where ER shows up much more often. Claessen et al. reported that ER appears about 50% more frequently in athletes than in people who aren’t involved in sports (OR 1.5 (SE 0.34), adjusted 95% CI 1.0 to 2.4). It also found a higher prevalence of 30% in the inferior leads (OR 1.3 (SE 0.38), adjusted 95% CI 0.74 to 2.3) and a 120% higher prevalence in the lateral leads (OR 2.2 (SE 1.0), adjusted 95% CI 0.87 to 5.4). Because of that, many experts, even those writing guidelines, often see it as a benign or physiological variant in this specific group [3].

When looking at the prognostic meaning of ER, most of the studies done in healthy people without clear structural heart disease suggest that ER doesn’t increase the risk of death from cardiovascular causes. That supports the idea that ER is mostly benign in the general population [5]. But things get more complicated when ER shows up in people with known or suspected arrhythmic conditions. For example, in patients with idiopathic ventricular fibrillation (IVF), ER has been linked to more frequent arrhythmia recurrences, which suggests that, in those cases, it might actually reflect something more dangerous [24].

Rath et al. reported that cohort studies have shown that around 32% of IVF patients have ER patterns. And those who do tend to have more frequent arrhythmic episodes, which is why it’s so important to take a closer look at ER after a person has had VF [24]. What makes it even trickier is that the ER in these cases isn’t always stable, and it can change over time. That dynamic nature makes risk prediction harder and points to the need for ongoing ECG checks in follow-up [25].

**Figure 5:**
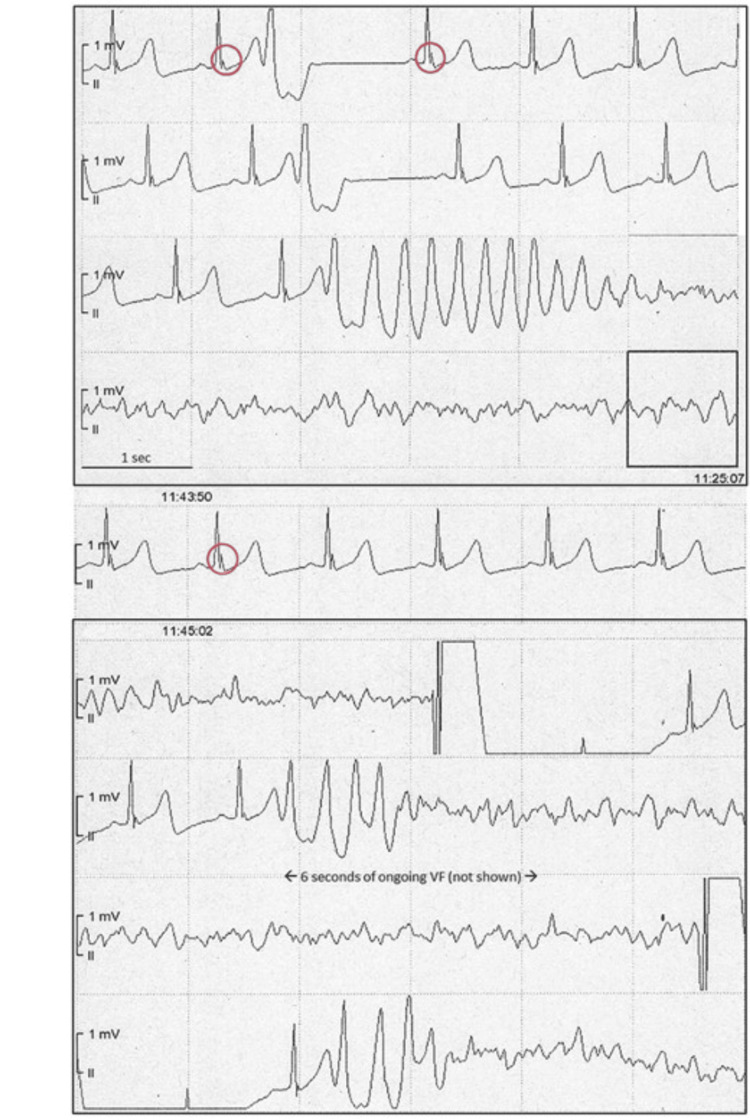
Continuous electrocardiographic tracings from a patient with electrical storm triggered by dynamic early repolarization Inferior J-waves with pause-dependent augmentation (red circles) precede the onset of ventricular fibrillation episodes, triggered by monomorphic premature ventricular complexes. Multiple defibrillations were required, and the patient eventually required VA-ECMO due to intractable VF. Figure reproduced from Steinfurt et al., licensed under CC BY-NC 4.0 [4]. VA-ECMO: venoatrial extracorporeal membrane oxygenation

This dynamic behavior of ER patterns in patients with IVF is illustrated in Figure 5. The continuous telemetry tracing demonstrates an electrical storm scenario where pause-dependent augmentation of inferior J-waves precedes the onset of polymorphic ventricular tachycardia, rapidly degenerating into ventricular fibrillation. The arrhythmic episodes are triggered by monomorphic premature ventricular complexes, with recurrence immediately after defibrillation, highlighting the malignant potential of certain ER subtypes in high-risk individuals [4].

There are a few ECG clues that may help distinguish between harmless and riskier forms of ER. The basic definition talks about J-point elevation of at least 0.1 mV in two or more contiguous leads, but many believe that the shape and direction of the ST segment matter even more. For instance, when the ST segment is horizontal rather than upward-sloping after the J-point, the risk seems to be higher. If the J-point elevation appears in many leads across the ECG, that also raises some concerns [24].

ER isn’t always acting alone. When it appears alongside other ECG abnormalities, the risk could go up. One example is when fQRS shows up with ER. This combo has been seen as a strong predictor of MACE, particularly in patients showing up with suspected acute coronary syndromes. This suggests there could be a kind of electrical instability happening on more than one level [15].

How ER behaves over time matters too. People who develop new-onset ER or who have persistent ER over multiple ECGs seem to be at greater risk of SCD and cardiovascular death in general. Patient follow-up over time and accurate representation of the ER in the patient's history are crucial [11].

Diagnostic approach

A comprehensive medical history and physical examination remain critical components of the initial evaluation of the ER. This assessment would include asking about any red flags that could indicate a higher risk for arrhythmia (history of palpitations, syncope, or family history of SCD) when the EKG indicates exercise-related rearrangement (examples of runners and weight-lifting training) [26,27]. On the other hand, ER in pediatric athletes appears to commonly result from physiological adaptations to training, is typically classified as a benign process, and is associated with very few major cardiovascular events during the follow-up [2].

One common component of the diagnosis, stress testing, is often used in athletes who may need to discover arrhythmias that only occur/exist during periods of exertion. Stress testing is especially valuable and should be viewed favorably when examining a resting ECG with mixed criteria as well as exercise-related symptoms [28]. Holter monitoring involves continuous ECG recording for 24 hours or more and may also be useful for the diagnosis of episodic and asymptomatic arrhythmias that a standard ECG recording may miss. Based on research from elite athletes, Holter recordings frequently demonstrate various findings such as bradycardia, sinus bradycardia, and occasional rare premature atrial or ventricular contractions. There were, however, very few major arrhythmias [29].

When there is a need for further investigation, advanced imaging modalities, such as echocardiography, are often performed to assess for potential structural problems that may also be causing the ECG pattern. However, it must be noted that in most cases, ER does not occur in patients with clear-cut structural heart disease, which is one of the distinguishing factors separating it from other pathologies [29].

In some instances, when non-invasive study findings show a reason for concern, for example, patients with recurrent syncope, documented arrhythmias, or family history, electrophysiological studies can be considered. Electrophysiological studies, while invasive, are the gold standard for diagnosing certain arrhythmic mechanisms and are typically only performed in individuals at high risk of SCD [28].

Management strategies

ER is seen frequently in athletes and is typically a benign variant of the athlete's heart, a grouping of physiological responses resulting from regular and sustained high-intensity physical activity that changes not only cardiac structure but also the electrical patterns without altering function [2,30]. Regarding pediatric athletes specifically, there remains a positive relationship between ER and clinical outcome. Large longitudinal studies have not reported any major cardiovascular events in pediatric athletes with ER after a median follow-up of 4.2 years, supporting the idea that ER represents a normal adaptation to training for pediatric athletes rather than an abnormal pathology [2].

In this context, patient education is paramount, as the information communicated to athletes and their families should reflect the general benign nature of ER (in the absence of clinical features or family history of SCD). That said, the utility of routine cardiovascular follow-up is important in all athletes, particularly for those in competitive sports activities, to monitor for any potential changes over time [30].

Although in the vast majority of cases, ER is highly reassuring, there are certain clinical circumstances that require further investigations. When palpitations or syncope occur in conjunction with ER or a family history of SCD is present, electrophysiology is warranted [31]. The presence of pacemaker-like behavior as well as other electrocardiographic diagnostic criteria of underlying heart disease, such as evidence of repolarization heterogeneity, conduction delay, or abnormal QRS form, also warrants follow-up [30]. The concern for arrhythmogenic conditions is particularly relevant to athletes who have an ER pattern that is atypical or related to a high-risk or prolonged endurance activity, as it may warrant additional assessment by electrophysiological studies [28].

As unlikely as it is, if ER coexists with severe bradycardia or arrhythmia and clearly impairs athletic performance, deconditioning or vagolytic therapies may be considered. These same mechanisms are thought to enhance vagal tone that may allow for higher levels of ER patterns in some individuals. It is worth noting that the lack of progress or resolution of symptomatology in enduring cases (not resolved by reassurance or standard follow-up) should only be a signal to exclude it as a form of treatment for ER [32].

Regarding sports participation, athletes with benign ER can, in most cases, continue with sports participation with no restrictions as long as they are asymptomatic and without a significant family history of SCD [33,34]. Cardiovascular screening before athlete participation is important from the standpoint of athlete safety. It helps identify athletes with particularly concerning findings prior to participation and to make more nuanced clinical decisions. Current guidelines recommend a strategy based on personalized risk assessment and shared decision-making with the athlete according to the athlete's context and needs [33,35]. 

In that respect, the European Society of Cardiology promotes the need to balance the well-established health benefits of regular exercise with rarer but possibly more serious cardiovascular risks. Their recommendations encourage open and informed discussion between athletes and clinicians as a fundamental part of safe participation, particularly in the case of conditions such as ER in the ECG [32].

Prognosis and follow-up

The implications of ER are currently being studied in different contexts, and the results seem to vary depending on the group of individuals being analyzed. In the general population, most of the evidence points toward ER not being a significant clinical concern. For instance, one study that followed 4,176 individuals without clear structural heart disease for 10 years didn’t find any meaningful link between ER and overall or cardiovascular-related mortality. According to their conclusions, ER didn’t seem to influence long-term outcomes in healthy people without a history of cardiac disease [5].

However, the picture becomes less clear when looking at middle-aged individuals. In a large cohort study with a median follow-up of 22.5 years, researchers found that time-varying ER, meaning new or changing ER patterns, was associated with a higher risk of SCD and cardiovascular mortality. The fact that ER can change over time, particularly in older adults, suggests that in some cases it might reflect a more concerning, arrhythmogenic condition. Based on this, the authors recommend ongoing follow-up to monitor how ER evolves in this population and what it might mean in terms of cardiovascular risk [11].

Among athletes, ER is far more common, with a prevalence that’s about 50% higher compared to non-athletes. This is especially relevant in young or pediatric athletes, where several longitudinal studies have shown that ER usually behaves in a benign way. In fact, no major cardiovascular events were reported over a four-year period in these younger individuals, which supports the idea that ER in this setting is more likely a normal adaptation to training rather than a pathological sign [2,3].

Holter monitoring has also been used to track heart rhythms in elite athletes with ER over a longer time span than a regular ECG. In most cases, it reveals patterns like sinus bradycardia or occasional premature atrial or ventricular contraction findings that are common in trained individuals and not typically dangerous. Serious arrhythmias were rarely observed, so Holter monitoring might not offer much value unless the athlete shows symptoms or has abnormal findings on a standard ECG [29].

To better understand the long-term outcomes of ER and other cardiac conditions in athletes, researchers created the Outcomes Registry for Cardiac Conditions in Athletes (ORCCA) study. This ongoing registry is designed to assess how athletes with heart conditions, including ER, fare over time. It uses a shared decision-making approach to determine whether or not someone is fit to continue participating in sports. The registry reflects a growing interest in tailoring risk assessments to the specific needs and circumstances of each athlete [36].

Finally, the Pro@Heart study is exploring how training intensity and genetic predisposition together shape cardiac remodeling and electrical patterns like ER. By examining how acute and chronic exercise, combined with specific genotypes, influence the heart’s electrical signals, this study hopes to offer new insights that could improve monitoring and risk assessment in athletic populations [37].

## Conclusions

The assessment of ER must be individualized based on age, prior clinical history, athletic status, and patient-specific variables. In young and athletic patients, ER may be a benign, physiological adaptation to physical training. Conversely, in individuals with older ages and personal or family histories of arrhythmia, dynamic ER patterns can carry an increased risk for SCD or any adverse cardiovascular events, requiring closer clinical evaluation.

Given the complex nature of ER, including morphology, stability over time, and other associated abnormalities, initial ER diagnosis can extend beyond a single ECG finding; therefore, assessment with other diagnostic modalities, including Holter monitoring and exercise testing, should be incorporated. Further evaluation with electrophysiological studies may also be appropriate even in higher-risk situations, as there may be a need to investigate the associated arrhythmia risk and expected subsequent action. Studies such as ORCCA and Pro@Heart have given a greater understanding of how to include genetic predisposition and even exercise training load to enhance risk stratification. Collectively, these factors can aid in determining the safe engagement of sport and overall cardiac risk management through collaborative, evidence-based clinical decision-making.
